# Genomic and proteomic characterization of SuMu, a Mu-like bacteriophage infecting *Haemophilus parasuis*

**DOI:** 10.1186/1471-2164-13-331

**Published:** 2012-07-23

**Authors:** Emilie S Zehr, Louisa B Tabatabai, Darrell O Bayles

**Affiliations:** 1 U. S. Department of Agriculture, Ruminant Diseases and Immunology, National Animal Disease Center, Agricultural Research Service, Ames, IA, 50010, USA; 2 U. S. Department of Agriculture, Infectious Bacterial Diseases, National Animal Disease Center, Agricultural Research Service, Ames, IA, 50010, USA

**Keywords:** *Haemophilus parasuis*, Bacteriophage, Virulence

## Abstract

**Background:**

*Haemophilus parasuis*, the causative agent of Glässer’s disease, is prevalent in swine herds and clinical signs associated with this disease are meningitis, polyserositis, polyarthritis, and bacterial pneumonia. Six to eight week old pigs in segregated early weaning herds are particularly susceptible to the disease. Insufficient colostral antibody at weaning or the mixing of pigs with heterologous virulent *H. parasuis* strains from other farm sources in the nursery or grower-finisher stage are considered to be factors for the outbreak of Glässer’s disease. Previously, a Mu-like bacteriophage portal gene was detected in a virulent swine isolate of *H. parasuis* by nested polymerase chain reaction. Mu-like bacteriophages are related phyologenetically to enterobacteriophage Mu and are thought to carry virulence genes or to induce host expression of virulence genes. This study characterizes the Mu-like bacteriophage, named SuMu, isolated from a virulent *H. parasuis* isolate.

**Results:**

Characterization was done by genomic comparison to enterobacteriophage Mu and proteomic identification of various homologs by mass spectrometry. This is the first report of isolation and characterization of this bacteriophage from the *Myoviridae* family, a double-stranded DNA bacteriophage with a contractile tail, from a virulent field isolate of *H. parasuis*. The genome size of bacteriophage SuMu was 37,151 bp. DNA sequencing revealed fifty five open reading frames, including twenty five homologs to Mu-like bacteriophage proteins: Nlp, phage transposase-C-terminal, COG2842, Gam-like protein, gp16, Mor, peptidoglycan recognition protein, gp29, gp30, gpG, gp32, gp34, gp36, gp37, gpL, phage tail tube protein, DNA circulation protein, gpP, gp45, gp46, gp47, COG3778, tail fiber protein gp37-C terminal, tail fiber assembly protein, and Com. The last open reading frame was homologous to IS1414. The G + C content of bacteriophage SuMu was 41.87% while its *H. parasuis* host genome’s G + C content was 39.93%. Twenty protein homologs to bacteriophage proteins, including 15 structural proteins, one lysogeny-related and one lysis-related protein, and three DNA replication proteins were identified by mass spectrometry. One of the tail proteins, gp36, may be a virulence-related protein.

**Conclusions:**

Bacteriophage SuMu was characterized by genomic and proteomic methods and compared to enterobacteriophage Mu.

## Background

*Haemophilus parasuis* causes Glässer’s disease in pigs, with symptoms of pneumonia, fibrinous polyserositis, pericarditis, polyarthritis, and meningitis [1]. *H. parasuis* can be isolated from the nasal passages of asymptomatic swine as well as those with septicemia and pneumonia without polyserositis. Due to lack of protective immunity to *H. parasuis,* introduction of conventionally raised pigs into segregated early weaning (SEW) herds may result in infection and high economic losses [2,3]. Losses in 2006 were approximately $145 million according to the National Animal Health Monitoring System (NAHMS) report [4] and Rodney B. Baker (Veterinary Diagnostic and Production in Animal Medicine, Iowa State University, Ames, IA, rbbaker@iastate.edu, personal communication).

*H. parasuis* comprises 15 serovars based on immunodiffusion assays which use heat-stable antigens [5]. Serovars 1, 5, 10, 12, 13 and 14 are considered highly virulent, resulting in piglet death within four days. Serovars 2, 4, 8, and 15 are moderately virulent, causing polyserositis but not death. Serovars 3, 6, 7, and 9 are considered avirulent and yield no clinical disease or lesions at necropsy. Many field isolates of *H. parasuis* are nontypeable (NT) [6].

The observed heterogeneity in bacteria are in part responses to environmental stress or host defense mechanisms, which select for deletions and rearrangements in the bacterial genome which can alter virulence and broaden host range [7]. Additionally, some heterogeneity among *H. parasuis* isolates is due to the acquisition of new DNA through bacteriophage transduction into the host chromosome [8], which is a common mechanism for bacterial genetic diversity. For example, approximately 50% of strain-specific genomic DNA of *Escherichia coli* O157 Sakai has been attributed to lateral gene transfer through bacteriophage sequence acquisition [8]. These results imply that bacteriophages may be instrumental in the emergence of new *E. coli* strains and contribute to the genomic diversity of the species.

*H. parasuis* serotype 5 has been described as a highly virulent strain. This report describes a bacteriophage, named SuMu, isolated from a field isolate of *H. parasuis* serotype 5. SuMu is a double-stranded DNA bacteriophage of the *Caudovirales* (tailed bacteriophage) order, *Myoviridae* family, and the Mu-like virus genus. SuMu is a bacteriophage that is related to enterobacteriophage Mu. Enterobacteriophage Mu is able to insert itself randomly via transposition at any point in the host chromosome and frequently causes mutations by interrupting the bacterial transcription at the site of the insertion. Mu-like bacteriophages are thought to carry virulence genes or can induce host expression of virulence genes [9,10]. A Mu-like bacteriophage gene of SuMu was associated with virulence in *H. parasuis*, the only swine bacteria that harbor Mu-like bacteriophages [11]. In this recent report [11], the Mu-like portal gene of bacteriophage SuMu was detected in 28 of 31 virulent field strains of *H. parasuis*. The SuMu bacteriophage was characterized here by DNA sequencing and proteomic identification of the bacteriophage proteins. DNA sequences were converted to proteins and protein homologies of enterobacteriophage Mu and bacteriophage SuMu were compared.

## Results

### Bacteriophage preparation

Bacteriophage was obtained from lysed cultures of *H. parasuis* field isolate 34086b [12] by a two-step centrifugation procedure. Because the time of lysis was variable, the cultures were allowed to “autolyse”, as determined by a decrease in optical density at 600 nm of 0.3-0.4 absorbance units, prior to harvesting at 26 to 48 h. Bacteriophage preparations contained approximately 2 x 10^4^ to 2 x 10^5^ PFU per ml of culture supernatant as determined by plaque assay.

### Electron microscopy

**Figure 1:**
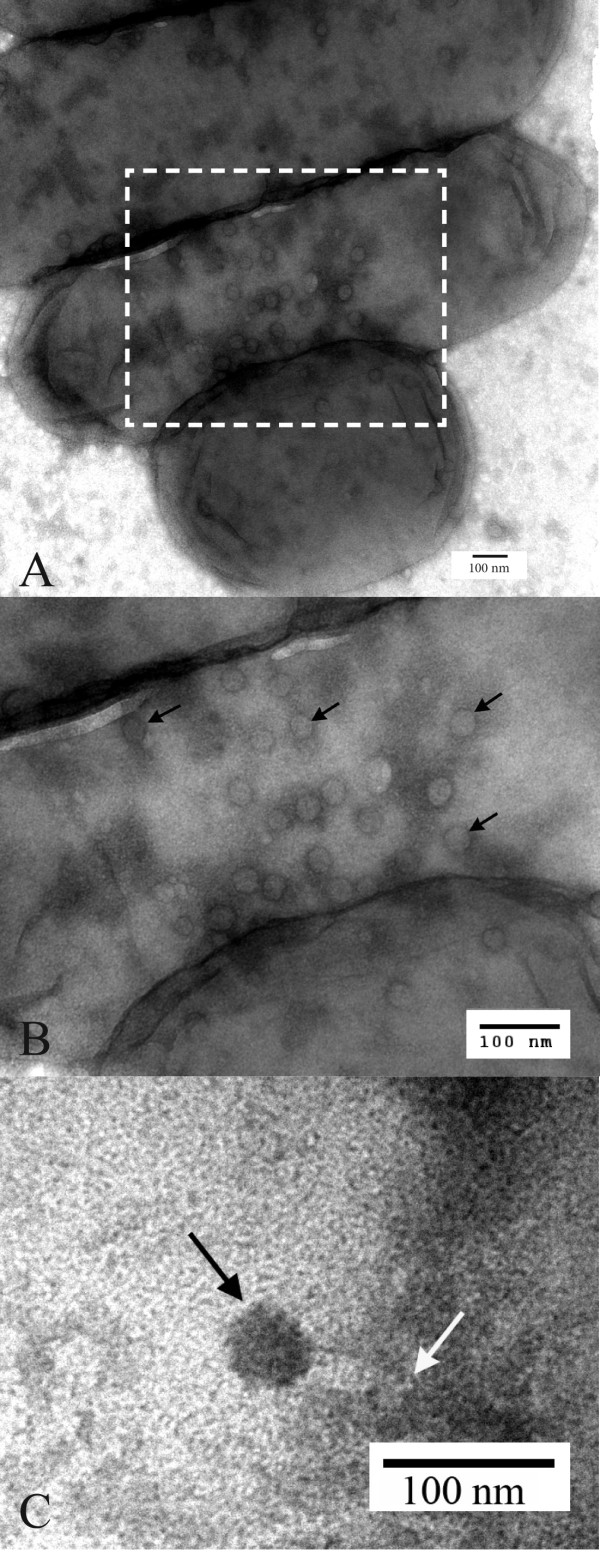
**Electron micrographs of bacteriophage SuMu associated with *H. parasuis* field strain 23806b showing (A) three bacteria from a 7 h lysate with attached icosahedral bacteriophage heads; (B) magnified (boxed) area of Figure 1A with icosahedral bacteriophage heads (black arrows); (C) an electron-dense DNA-filled bacteriophage head (black arrow) and tail sheath (white arrow) attached to a bacterium from an overnight lysate.**

Electron microscopy was used to determine bacteriophage morphology. Electron micrographs of bacteriophage associated with *H. parasuis* field strain 23806b are shown in Figure 1. The digitally measured average icosahedral head width was 42.1 +/−2.1 nm and the average tail sheath length was 49.2 +/−6.6 nm. Figure 1A shows a 7 h lysate of three bacteria with many icosahedral bacteriophage heads attached. A dashed box outlines a portion of Figure 1 which is magnified in Figure 1B. Black arrows identify selected icosahedral bacteriophage heads in Figure 1B. An electron-dense DNA-filled head (black arrow) and tail sheath (white arrow) attached to bacteria from an overnight lysate are shown in Figure 1CC. Refractive indices (η) of the collected cesium chloride gradient bands were between 1.3792 and 1.3802, which correspond to densities of approximately 1.47 g/ml. Essentially no bacteriophages were seen by electron microscopy after the cesium chloride step gradient centrifugation procedure. This may have partially been due to background problems.

### Cloning and DNA sequencing

The genomic sequence of SuMu was obtained from a single cloned fragment of purified bacteriophage DNA and represents the Mu-like bacteriophage in *H. parasuis* 34086b. From approximately thirty spin-column purified clones, only three contained inserts. All three inserts were submitted for DNA sequencing. The first insert was 700–800 bp in length and its peptide had 32% identity over 91 amino acids to Mu I (Accession No. NP_050636). The other two inserts were identical and were 461 bp in length. Their peptides had 30% identity over 36 amino acids to Mu gp29 (Accession No. AAF01107.1).

Ultracentrifuged, amplified bacteriophage was used as additional template for genomic DNA. The bacteriophage genome was sequenced by using overlapping primers to previously sequenced DNA and primer walking with 2- to 15-fold coverage of the DNA. Sequencing through variable terminal repeats was accomplished by redesigning primers and sequencing unique PCR segments of bacteriophage DNA that could be inserted into the genome assembly. A homolog to IS1414 with a MULE transposase domain was found at CDS 36941..37151. Inverted repeats were located at bp 294 (ATTTTGCATAGCA) and bp 319 (TGCTATGCAAAAT); bp 36815 (GGTTTTTAAAT) and bp 36840 (ATTTAAAAACC); bp 36942 (AAAAAATGCGGT) and bp 36976 (ACCGCATTTTTT). Direct repeats were located at bp 35956 and bp 36069 (TTTTTAAAGTTA).

The G + C content of the bacteriophage was 41.87%, while the G + C content of its *H. parasuis* 34086b host bacteria was 39.93%. The genome size of *H. parasuis* bacteriophage was 37,151 bp. Significant portions of the bacteriophage SuMu and enterobacteriophage Mu DNAs were dissimilar. Only 43.7% of their DNA sequences matched and overall there was 50.2% amino acid homology over 3,733 amino acids. Additionally, a partial hemolysin gene (302 bp) (*E*-value = 0.0) was detected 2,478 bp upstream of the putative SuMu repressor gene (CDS 353..1072 c).

### Protein homologies of bacteriophage SuMu

**Figure 2:**
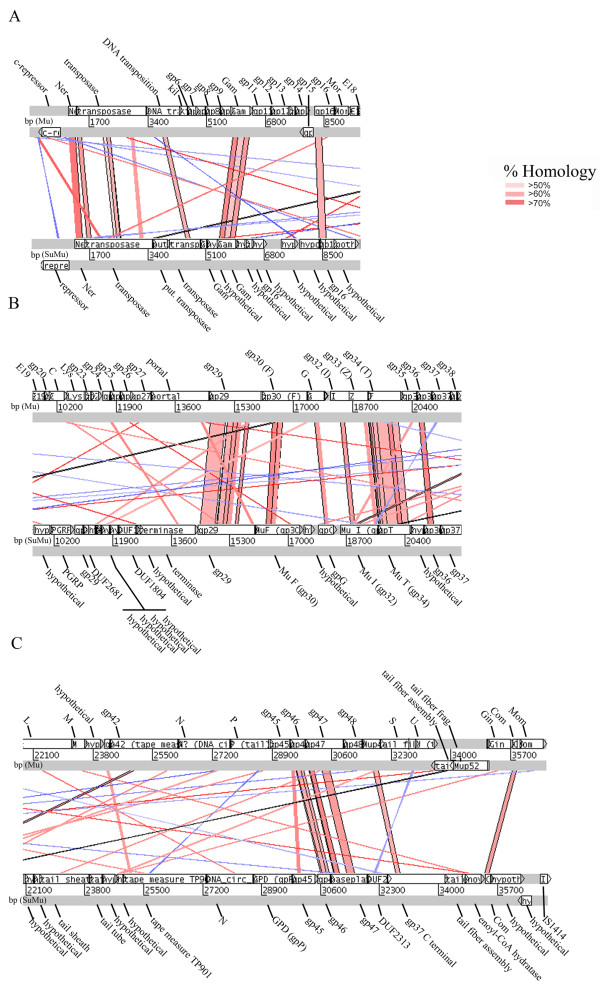
**Comparison of the bacteriophage SuMu DNA (lower sequence) to enterobacteriophage Mu DNA (upper sequence) using ACT: Artemis Comparison Tool [**[16]**].** Open reading frames are shown in white boxes under each DNA sequence and are labeled for each genome. Panel A shows CDS 1..9447, Panel B shows CDS 9617..22056, and Panel C shows CDS 22053..37151 of bacteriophage SuMu with the corresponding open reading frames in enterobacteriophage Mu. Areas of amino acid homology (forward sequence matches) found with tblastx (Search translated nucleotide subjects using a translated nucleotide query) are shown in shades of red. Homologies range from greater than 50% to greater than 70%. Black lines indicate the edges of blast matches. Areas with reverse sequence matches are shown in blue.

Fifty four potential coding regions of bacteriophage SuMu and one insertion sequence were identified by using tblastx analysis [13] (Figure 2, Panels A-C). Seventeen of the potential coding regions were homologous to enterobacteriophage Mu proteins, as shown by the bars in shades of red connecting the respective bacteriophage genes (Figure 2, Panels A-C, Additional file 1; Table S1). Scattered lines (Figure 2, Panels A-C) represent homologies that are not within genes but which indicate additional recombination events between the two bacteriophages. Twenty five of the 54 potential proteins were homologs of Mu-like proteins using NCBI’s Open Reading Frame Finder mining tool [14] (*E*-values < 1e^-16^ by blastp analysis) [15] (Additional file 2; Table S2) .

The comparison between bacteriophage SuMu and enterobacteriophage Mu proteins (Figure 2 and Additional file 1; Table S1) revealed homologies with transcription regulators and transposases (*E*-values from 4e^-13^ to 5e^-24^) as well as high homologies (*E*-values from 7e^-30^ to 3e^-89^), with the host-nuclease inhibitor protein Gam and structural proteins, including portal protein (gp29), Mu F (gp30), Mu I (gp32), gpT (gp34), and gp47. There was low homology (*E*-values from 1e^-06^ to 2e^-15^) to gp16, gpG, gp36, baseplate assembly protein (gp45), gp46, and Com, a translational regulator of Mom. There was negligible homology (*E*-values 0.53 and 0.004, respectively) to the SuMu tape measure protein TP901 and to the tail fiber protein (gp49).

The comparison of bacteriophage SuMu to Mu-like bacteriophages by blastp analysis (Additional file 2; Table S2) showed greater amino acid homologies than there was to the Mu enterobacteriophage. *E*-values were higher and more proteins of Mu-like bacteriophages were homologous to bacteriophage SuMu than there were to enterobacteriophage Mu. Overall bacteriophage SuMu had 96.9% homology over 6,786 amino acids of the other Mu-like bacteriophages.

### Protein analysis by mass spectrometry

One- and two-dimensional electrophoresis of the TCA-treated bacteriophage preparation and BHI medium-only control sample were processed by mass spectrometry. The twenty proteins identified based on the masses of their tryptic fragments are listed in Additional file 3; Table S3. All of the bacteriophage protein homologs were from bacteriophages in the *Myoviridae* family except for two tape measure proteins from the *Siphoviridae* family. Only one of the 11 mass spectra submitted to PRIDE was identified as a peptide (accession number 22479).

Fifteen of the 20 bacteriophage proteins identified by mass spectrometry are homologs of structural bacteriophage proteins according to blastp analysis [15]. A total of 11 bacteriophage tail structural protein homologs were identified; among these were the bacteriophage tape measure protein, which functions in determination of bacteriophage tail length (homologs 1 (NP_463477), 2 (YP_239811) and 11 (P44236), respectively). Homolog 9 (YP_398561) is a transglycosylase related to the bacteriophage tape measure protein, TP901 family. The bacteriophage protein gp37, homologous to the long tail fiber receptor recognizing protein [homolog 3 (P03744)], allows the specific attachment of the bacteriophage to the bacteria and specifies the host range of the bacteriophage. Other bacteriophage proteins, homologs involved in attachment to the host cell’s lipopolysaccharide include the tail fiber protein, and gp36, respectively (homologs 6 (YP_249019), 7 (P44242), and 20 (P03743), respectively). The bacteriophage tail sheath protein (gpL), homologs 4 (YP_0010604090) and 5 (YP_004324195), may form the outer contractile sheath of the capsid tail. The baseplate wedge protein homolog 14 (P10927) may connect the tail fiber to the baseplate and trigger tail contraction after virus attachment to the host cell. The portal protein (gp29), protease (gp32), major head protein (gp34), Mu F protein (gp30), homologs 10 (P44225), 13 (NP_873073), 15 (P44227), and 17 (ZP_00157240), respectively, are involved in bacteriophage morphogenesis [10].

Two proteins were similar to proteins in the NCBI database which were putatively associated with lysogeny and lysis functions of other bacteriophages, namely Ner protein and muramidase. The DNA binding protein Ner regulates transcription of the genome of enterobacteriophage Mu [homolog 18 (P46496)]. The muramidase, homolog 19 (Q9T1X2), may be involved in host cell lysis. Three proteins (homologs 8, 12, and 16) are probably involved in integration and replication of the bacteriophage: transposase A (O05069), prophage integrase (EGT81757), and DNA transposition protein B (P96343), respectively. Thirteen of the proteins identified by mass spectrometry were also found in blast searches for protein homologies to DNA sequences between bacteriophage SuMu and enterobacteriophage Mu (Additional file 1; Table S1) or other Mu-like bacteriophages (Additional file 2; Table S2).

### Bacteriophage SuMu sequences in *H. parasuis* 34086b genomic DNA compared to the Nr database

Potential SuMu-like sequences of *H. parasuis* 34086b were compared to the NCBI non-redundant (Nr) database using the blastn algorithm in order to determine if there were matches to the bacteriophage SuMu sequences. *H. parasuis* 34086b genomic DNA segments were homologous to the following bacteriophage SuMu DNA segments with *E*-values of 0.0: partial hemolysin-transposase (CDS 114..2622), transposase-hypothetical protein 9 (CDS 2662..6047), hypothetical protein 25-DUF1804 (CDS 12661..14174), DUF1804-terminase (CDS 12161..14176), gp29 (CDS 14605..15665), gpG-gp36 (CDS 18192..21273), DNA circulation protein N-gp37 C-terminal protein (CDS 28500..32761). *H. parasuis* 34086b Mu-F-like DNA (CDS 16319..17132) and IS1414 (CDS 36896..37151) were also homologous to bacteriophage SuMu DNA, with *E*-values of 2e^-74^ and 9e^-123^, respectively.

**Putative rearrangement of SuMu prophage DNA in *H. parasuis *34086b.** The chromosomal Mu-like sequences were discontinuous except for one putative prophage SuMu sequence. The sequence of the *H. parasuis* 34086b DNA revealed a putative rearrangement of DNA involving the prophage. There was a deletion of the prophage SuMu genes gp37-C-terminal through its end at IS1414 (CDS 32762..37151). Another deletion of 6121 bp occurred between hypothetical protein 9 and DUF1804 of prophage SuMu (CDS 6046..12168), where an IS1414 element was inserted.

## Discussion

The study reported here describes the genomic and proteomic analysis of a bacteriophage isolated from a virulent field strain of *H. parasuis*. The genome size of the *H. parasuis* bacteriophage (SuMu) is 37,151 bp and is comparable to the genome sizes of enterobacteriophage Mu of *E. coli* K-12 (36,717 bp), *H. influenzae* bacterio-phage FluMu (34,676 bp), and the *Neisseria meningitidis* Mu-like bacteriophage Pmn1 (39,236 bp) [10,17] .

Bacteriophage SuMu’s morphology is similar to amber mutants of enterobacteriophage Mu described by Grundy and Howe [18]. An icosahedral head and tail sheath were seen in bacteriophage SuMu by electron microscopy. Our lysates were stored at 4°C for over a week before electron microscopy. Few intact virions for this bacteriophage were found probably due to their disintegration upon storage. These findings are in agreement with those of Summer et al. [19], who found that phage 56 (BcepMu) particles were unstable in lysates, had decreasing titers with storage, and showed disintegrating particles with broken heads, and partially exposed tails in electron micrographs.

Although bands were observed after cesium chloride centrifugation at the refractive indices (η) reported by Grundy and Howe [18], no bacteriophages were detected by electron microscopy. Our SM broth had 50% less concentrations of Tris–HCl and magnesium than the final buffer used by Grundy and Howe [18]. We also removed the cesium chloride by ultrafiltration rather than by dialysis. These methods may have also affected the stability of the bacteriophages. However, our electron micrographs clearly showed bacteriophage particles attached to the bacteria in Figure 1 and Figure 1B.

The G + C content of the *H. parasuis* bacteriophage SuMu at 41.87% is similar to the G + C content of the virulent *H. parasuis* (39.93%). This close match is also seen in the G + C contents of the Mu-like bacteriophage Pmn1 (53.1%) and its host genome *N. meningitidis* type A strain Z2491 (51.8%), and enterobacteriophage Mu (52.05%) and the *E. coli* K-12 genome (50.8%) [10,20]. In contrast, the G + C content of *H. influenzae* Mu-like bacteriophage FluMu is approximately 50%, compared to 38% G + C of the *H. influenzae* Rd chromosome [10].

A comparison of the bacteriophage SuMu translated nucleotide sequence obtained from its cloned DNA to the enterobacteriophage Mu translated nucleotide sequence using the Artemis Comparison Tool (ACT) revealed that the conserved genes generally were related to lysogeny and lysis, integration and transposition, morphogenesis, and tail proteins. Interestingly, similar bacteriophage sequences were also present in two published *H. parasuis* genomes. Thirty nine of the 55 bacteriophage SuMu genes were homologous to DNA sequences from *H. parasuis* strain 29755 (GenBank NZ_ABKM00000000, ctgs_1000001-1000246) or *H. parasuis* SH0165 [21]. Bacteriophage genes were also reported as upregulated by Melnikow et al. [22] using a microarray analysis of the *H. parasuis* genome after growing *H. parasuis* strain 29755 under iron-limiting, oxygen-limiting, heat, and acidic conditions. The highest gene homologs found were bacteriophage transposase (DQ127936; 99% identity over 1,043 bp; *E*-value = 0.0), bacteriophage Mu T protein homolog (DQ127950; 96% identity over 924 bp; *E*-value = 0.0), and bacteriophage Mu I protein GP32 (DQ127939; 97% identity over 1,077 bp; *E*-value = 0.0).

The partial hemolysin gene found upstream of the SuMu putative bacteriophage repressor gene (CDS 353..1072 c) was 99.3% similar (over 302 bp) to *hhd*B, which encodes a hemolysin activation/secretion protein (NC_118252, 1417483..1419203) of *H. parasuis* SH0165, serovar 5. Others have reported a putative hemolysin gene operon, *hhdBA*, in pigs infected with *H. parasuis*[23,24]. The bacteriophage SuMu partial hemolysin gene was probably a remnant of the *H. parasuis* 34086b host chromosome, which could have been acquired by an illegitimate recombination event.

However, the partial hemolysin gene homolog was not found in the *H. parasuis* draft 29755, serovar 5 genome (Genbank NZ_ABKM00000000, ctgs 1000001–10000246). Given the draft nature of the *H. parasuis* 29755 genome, it is possible that assembly breaks, which are not uncommon at bacteriophage and repetitive sequences, may have prevented the annotation of a hemolysin gene if one is present.

The bacteriophage SuMu protease contained a significant deletion (254 amino acids) compared to the Mu I bacteriophage protease. The other two initial inserts of cloned DNA had deletions of 127 amino acids when they were compared to the Mu gp29 sequence.

As has been reported in the literature [10,25-27], a bacteriophage may contribute to the virulence of its host. Avian pathogenic *E. coli* causes respiratory infection and septicemia of poultry, involving bacteriophage-related sequences coding for proteins such as DNA stabilization, portal, and integrase proteins [26]. Bacteriophage SuMu gp29 and gp36 are a homologs of enterobacteriophage Mu gp29 and gp36, found in *H. influenzae* Rd [10]. SuMu gp29 and gp36 are also homologous to two genomic fragments identified by Townsend et al. [25] which hybridize to virulent hemorrhagic septicemia isolates of *P. multocida*[27], but not to other *P. multocida* isolates. Translated DNA of one fragment (clone 6b) of *P. multocida* identified by Townsend et al. [25] had 62% homology [28] over 87 AA to SuMu_34, similar to gp36 of enterobacteriophage Mu (*E*-value = 1e^-17^) by blastp analysis [15]. The other translated DNA fragment (clone A3b) [25] had 72% homology over 83 AA to SuMu_27, similar to enterobacteriophage Mu gp29 (*E*-value = 4e^-31^). SuMu proteins may also be associated with virulence in swine infected with *H. parasuis* carrying the SuMu bacteriophage [29].

Canchaya et al. [30] have reviewed work on lysogenic conversion. Some bacteriophages carry DNA that can alter the phenotype of the bacterial host (lysogenic conversion genes) and this lysogeny is a form of short-term bacterial evolution. When lysogenic Gram-negative bacteria were grown in animals, the bacteriophage-specific genes were upregulated in the bacteria [26]. This study described Mu-like genes that mirrored those found in enterobacteriophage Mu and MuMenB, a bacteriophage of *N. meningitidis* strain MC58 [17]. Corresponding Mu-like genes found in Mu, MuMenB and SuMu (reported here) included Mu A, Mu B, gp29, gp30, Mu G, Mu I, gp36, gp37, gpL, Mu M, gp42, gp45, gp46, and gp47. Two strains of *H. parasuis*, strain 29755 [31] and strain SH0165 [21], whose genomes have been sequenced, also carry Mu-like bacteriophages. The Mu-like bacteriophage GenBank NZ_ABMK00000000, ctg_1000002 sequence of strain 29755 is similar to that of the SuMu DNA sequence. The tail assembly protein of *N. meningitidis* (Mu G) has been postulated to be membrane-associated. In these studies [17], the authors hypothesized that bacteriophage-encoded membrane-associated proteins of *H. influenzae* and *N. meningitidis* contributed to the variability of the bacterial envelope structure and may therefore influence the virulence and pathogenicity of the organisms.

DNA sequencing revealed a peptidoglycan recognition protein (PGRP) which hydrolyzes peptidoglycans of bacterial cell walls at the analogous position of the lysis protein of enterobacteriophage Mu. Mass spectrometry also suggested a lysozyme/muramidase/endolysin.

The mass spectrometry analysis of the proteins from the 2-D gel suggested that *H. parasuis* strain 34086b may contain more than one bacteriophage as was shown by others. For example, *H. parasuis* strain SH0165 [21] also carries Lambda, P2, and CP-933 K bacteriophage genes while strain 29755 [31] has remnants of Lambda, CP4-57-like, P4-like, phi-C3, CP-933 C, and Lj965 bacteriophage genes. However, the SuMu genome sequence was based on cloned DNA and contained only one bacteriophage.

Bacteriophage SuMu was putatively distantly related to the enterobacteriophage Mu by combined DNA and proteomic evidence. Only 17 out of 54 putative proteins were more than 50% homologous between enterobacteriophage Mu and bacteriophage SuMu. The percent homology and *E*-values associated with them when comparing their translated nucleotides and their proteins was low compared to similar comparisons of bacteriophage SuMu to other Mu-like bacteriophages. These results are in agreement with the evolutionary diversity of bacteriophages [32,33]. Although the double-stranded DNA tailed-bacteriophages are extremely diverse, they share common modules, such as terminase and portal proteins as well as tail proteins [32]. Either by illegitimate recombination or by integrase-mediated site specific recombination, a structural gene operon can be changed so it is not homologous to other genes in related bacteriophages [33]. The presence of variable terminal sequences in bacteriophage SuMu supports the classification of it as a Mu-like bacteriophage. The rearrangement of SuMu prophage sequences in *H. parasuis* 34086b is corroborated by a report of CampMu prophages mediating genomic rearrangements in *Campylobacter jejuni*[34]. Since bacteriophage SuMu’s G + C content is not much different than its host’s chromosome G + C content, it can be concluded that SuMu has been associated with *H. parasuis* for a relatively long length of time [10].

Based on the results of others [27,30,35], it is suggested that some virulent bacteria harbor bacteriophages while many avirulent organisms do not have genome-associated bacteriophage. Our recent study [11] showed the distribution of SuMu’s Mu-like portal bacteriophage gene, gp29, among 15 reference strains and 31 field isolates of *H. parasuis*. The gene was present in most of the virulent isolates and absent in most of the avirulent isolates. This nested PCR assay detected 28 of 31 field isolates designated as virulent and five of six reference strains designated as virulent by utilizing the gp29 gene of bacteriophage SuMu.

Since the bacteriophage SuMu genes and proteins identified are related to bacteriophage Mu, they could potentially confer the necessary factors for this bacteriophage to be able to transduce virulence factors, which could affect the epidemiology of *H. parasuis* field isolates [9,10]. A potential diagnostic test such as a bacteriophage-specific PCR test was developed to assay for the presence of bacteriophage genes in bacterial isolates from *H. parasuis*-infected animals [11].

## Conclusions

In summary, this is the first report on the characterization of a bacteriophage of a virulent field strain of *H*. *parasuis* named SuMu (Accession No. JF832915) by DNA sequencing and by 1-D and 2-D SDS-PAGE electrophoresis of bacteriophage proteins, followed by mass spectrometry. Fifty five open reading frames were identified, correspon-ding to fifty four putative bacteriophage SuMu proteins, seventeen of which were related to enterobacteriophage Mu and twenty five of which were related to other Mu-like bacteriophages. The last open reading frame in bacteriophage SuMu corresponded to IS1414. Proteomic technologies identified twenty homologs of bacteriophage proteins. The combined DNA and proteomic evidence suggests that bacteriophage SuMu and the enterobacteriophage Mu are putatively distantly related. It appears that this Mu-like bacteriophage of *H. parasuis* may code for at least one potential bacterial virulence factor (gp36) which may contribute to septicemia in swine [25,29]. Since the bacteriophage SuMu genes and proteins identified are related to enterobacteriophage Mu, they could potentially confer the necessary factors for this bacteriophage to be able to transduce virulence factors [9,10], which could affect the epidemiology of *H. parasuis* field isolates.

## Methods

### Strain and growth conditions

*H. parasuis* field strain 34086b was isolated from systemic sites (lung and stifle joint) of a clinically affected pig (Lorraine Hoffman, Iowa State University, Veterinary Diagnostic Laboratory, Ames, IA, personal communication). The specimen was protected from drying and cultured within 24 h after collection. Two 5% sheep blood agar plates and one 4% sheep blood agar plate were inoculated and streaked for isolation. One 5% blood agar plate and one 4% blood agar plate were then cross streaked with *Staphylococcus spp*. These plates with the nurse streak were incubated at 35-37°C in 5-10% CO_2_ environment. The second 5% blood agar plate was incubated at 35-37°C in an anaerobic jar. A chocolate agar II plate (Becton, Dickinson and Company, Sparks, MD) was incubated at 35-37°C in 5-10% CO_2._ All plates were incubated for a total of 48 h before calling the specimen negative.

After 24–48 h incubation, small dewdrop-like colonies that were consistently nonhemolytic and tiny nonhemolytic colonies that were satellites along the nurse steak supplying the β-NAD were tentatively identified as *H. parasuis*. Microscopically, the colonies were Gram-negative rods that might also be coccobacillary in form. Biochemically, the isolate was urease negative.

In our laboratory, the bacteria were propagated in Frey’s mycoplasma base broth (Sigma, St. Louis, MO) modified by supplementation with 20% heat-inactivated horse serum (Invitrogen, Carlsbad, CA) and 0.016% β-NAD (Sigma) (Vicki Rapp-Gabrielson, personal communication) and incubated overnight at 37°C. Among 31 field strains obtained from the Iowa State University Veterinary Diagnostic Laboratory, field strain 34086b appeared to lyse overnight broth cultures while most of the other isolates did not. Purity of the cultures was confirmed by preparing a lawn of culture on trypticase soy agar base plates with 5% defibrinated ovine blood (Becton, Dickinson and Company) and streaking a “nurse” or feeder streak of *Staphylococcus aureus* as a source of β-NAD across a lawn of the *H. parasuis* isolate to be tested. Purity was also tested by colony appearance on Casman’s agar (Difco, Detroit, MI) containing 5% horse serum and 0.016% β-NAD (Vicki Rapp-Gabrielson, personal communication) [36]. Cultures were incubated at 37°C under humidified 5% CO_2_. Field strain 34086b was serotyped by the immunodiffusion method using heat-stable antigen extracts [5] by Gallant Custom Laboratories (Cambridge, Ontario, Canada) as serotype 5.

### Isolation of bacteriophage

The procedure of Williams et al. [37] was followed to isolate the bacteriophage. Briefly, *H. parasuis* field isolate 34086b (serovar 5) was grown in brain heart infusion (BHI) broth (Difco/Becton Dickinson) supplemented with 10 μg/ml heme-HCl (Fluka), 10 μg/ml L-histidine (Sigma), 0.16 mg/ml ß-NAD, and 10 mM magnesium sulfate. Overnight cultures (10 ml) were inoculated into 100 ml supplemented BHI in 250 ml Erlenmeyer flasks; media and flasks were rotated at 75 rpm on an orbital shaker at 37°C. Cultures were allowed to autolyse and release the bacteriophage and were harvested at 26–48 h post-inoculation. The extent of lysis was monitored by measuring the decrease in absorbance of the culture at 600 nm. Cultures were harvested and centrifuged at 20,000 × *g* for 15 min at 4°C. The pellet was discarded and the supernatant was recentrifuged, then filtered through a 0.22 μm filter (Millipore) and 2 ml of chloroform per 100 ml of supernatant was added. The supernatant was concentrated by centrifugation at 145,000 × *g* for 3 h. The pellet was resuspended in a minimal amount of 50 mMTris–HCl, pH 7.8, and containing 10 mM MgSO_4_ (TM) buffer. The resuspended pellet was dialyzed against TM buffer for 2 h and filtered as above. The bacteriophage was stored at 4°C.

### Plaque assay

The supernatant of field strain 34086b bacteriophage preparation, obtained after centrifugation at 20,000 × *g*, was serially diluted in Frey’s medium supplemented with ß-NAD. One ml of each dilution was removed to sterile tubes containing 0.064 ml 1% ß-NAD and 0.5 ml horse serum. One-tenth ml overnight culture of *H. parasuis* cells was added; the mixture was vortexed and incubated for 40 min at 37°C. Three ml of 0.4% SM top agarose was added [38], the tube was inverted to mix, and poured onto a Casman’s agar plate. The agarose was allowed to solidify and an additional 3 ml SM top agarose was added. Plates were incubated upright in 5% carbon dioxide atmosphere at 37°C and formation of plaques was recorded after 15–18 h of incubation [38].

### Electron microscopy

For bacteriophage electron microscopy, low passage *H. parasuis* field strain 23086b serovar 5 bacteria were collected at 7 h post-passage. An aliquot (1.5 ml) was removed and centrifuged at 4°C for 5 min at 400 × g. The supernatant was discarded and the pellet was washed in 100 μl of 5 mM PBS; then resuspended in 50 μl of 5 mM PBS. For some preparations, lysates were stored at 4°C for one week before electron microscopy. The bacteriophage/bacteria preparation (10 μl) was mixed 1:1 with 2% phosphotungstic acid (pH 7.0) for 3 min on a carbon-coated grid, the excess fluid was removed by filtration, and the grid was viewed on a Technai 12 transmission electron microscope (FEI Company, Hillsboro, OR). Structure sizes were determined by marking them as they were viewed on the electron microscope.

Bacteriophage from 250 ml of culture lysates were also purified by after addition of sodium chloride and solid polyethylene glycol 6000 (Sigma), followed by centrifugation in step gradients of cesium chloride, as described by Maniatis et al. [38]. Bands were collected, concentrated and washed twice with 0.5 ml SM broth containing 50 mM Tris–HCl, 0.8 mM magnesium sulfate, 0.1 M sodium chloride, and 2% gelatin [38] by ultrafiltration at 10,000 × *g* using a centrifugal ultrafilter with a MWCO of 100,000 (VWR, Batavia, IL). The bands were analyzed by electron microscopy as above except they were incubated 1:1 in 2% phosphotungstic acid for 5 min, the grid was turned upright for an additional 1 min, and then the excess fluid was removed by filtration.

### Cloning, DNA sequencing, and data analysis

Fifty μl of dialyzed bacteriophage preparation obtained by centrifugation at 145,000 × *g* was treated with 0.5 units each of DNase and RNase for 30 min at 37°C, then 20 units of proteinase K (BRL) and 1% SDS for 1 h at 65°C. The sample was extracted with phenol three times [38] and precipitated with ethanol. The DNA was resuspended in 50 μl 10 mM Tris–HCl, 1 mM EDTA, pH 8.0. The bacteriophage DNA was amplified using a GenomiPhi DNA amplification kit (Amersham Pharmacia Biotech, Piscataway, NJ). The amplified product was heat-inactivated and precipitated with ethanol containing 0.15 M sodium acetate, 25 mM EDTA, reconstituted in buffer EB (Qiagen, Valencia, CA), and separated by electrophoresis on a 0.6% gel at 100 V for 60 min. The size of the amplified DNA was compared to amplified Lambda kit control DNA, Lambda DNA (New England Biolabs (NEB), Ipswich, MA), and *Hin*d III-digested Lambda DNA (NEB). The latter two standards were preheated to 60°C for 3 min prior to loading on the gel.

The amplified DNA was resolved by electrophoresis on a 0.8% agarose gel and DNA fragments between 2–23 kb were eluted from the gel using a Qiaquick kit (Qiagen). The gel-extracted bacteriophage fragments were cloned into pCR2.1 TOPO XL (Invitrogen) and transformed into chemically competent *E. coli* bacteriophage-resistant cells (One Shot Mach1-T1^R^) (Invitrogen) using manufacture-recommended conditions (Invitrogen). Transformed colonies were picked and grown in 5-ml overnight cultures. Plasmid DNA was isolated using a QIAprep spin plasmid miniprep kit (Qiagen).

Automated dideoxy sequencing [39] of *Bam*HI/*Not*I inserts was performed at the Iowa State University DNA Sequencing and Synthesis Facility, which used the Applied Biosystems (Foster City, CA) Prism BigDye Terminator v3.1 cycle sequencing kit with AmpliTaq DNA fluorescent sequencing (FS) polymerase. The reactions were separated by electrophoresis on an Applied Biosystems 3730 DNA analyzer. The bacteriophage genome was sequenced by primer walking after initial primers were generated from the cloned inserts and from sequence analysis of the *H. parasuis* 34086b prophage genome. The ends of the bacteriophage genome were sequenced by designing multiple primers that were originally homologous to insertion sequences of *H. parasuis* SH0165 ISax1 and IS1414. These primers were used to generate unique PCR products that closed the gaps in the bacteriophage DNA and these PCR products were then sequenced for use in the assembly of the genome. DNA sequences were assembled and analyzed with Lasergene version08 software (DNASTAR Inc., Madison, WI). Other DNA analysis tools were ExPASy [40] where translated DNA sequences were converted to protein sequences; Bioedit [41] and Dialign 2 [42], which are DNA sequence alignment tools; blastn [13], which searched a nucleotide database using a nucleotide query; and ORF (Open Reading Frame) Finder (updated April, 2011) [14], which found all ORFs in our DNA sequence in standard or alternative genetic codes. The ORFs were analyzed using blastp [15] (protein database 2.2.24+ was searched using protein queries).

The bacteriophage SuMu DNA sequence was compared to the enterobacteriophage Mu DNA sequence using ACT: Artemis Comparison Tool [16]. The ACT comparison tool used the tblastx program which searches a translated nucleotide database using a translated nucleotide query. The parameters used in the tblastx search were –f 999 which specified a high threshold for extending word hits. We decreased the search sensitivity here so that we would not get insignificant homologies. The –F mS parameter specified the filter used to mask the query sequence and masked the lookup table only without affecting the extension. The –e 100 parameter specified the expectation value cutoff and controlled the specificity. Less significant hits have settings greater than 10.

### Isolation and sequencing of *H. parasuis* 34086b genomic DNA

7Strain *H. parasuis* 34086b was cloned three times. Genomic DNA was prepared from the third clone according to the instructions of the Wizard Genomic DNA Purification Kit (Promega Corporation) with the following modifications: Eight ml of *H. parasuis* 34086b cells with an optical density of 0.9655 at 750 nm were pelleted successively into one 2 ml microcentrifuge tube at 16,000 × g. The cells were resuspended in 480 μl of 200 mM EDTA, 60 μl of 10 mg/ml lysozyme, and 60 μl of double distilled water. The genomic DNA was resuspended in 100 μl Buffer EB (Qiagen, Inc.). Genomic DNA was quantified using Quant-iT PicoGreen (Invitrogen, Carlsbad, CA) and 5 micrograms of DNA was used in the Titanium General Library Preparation method (454 Life Sciences, Branford, CT). The resulting library was subjected to emPCR and used to prepare DNA beads for sequencing using a single region of a four region picotiter plate on a Roche GS-FLX instrument using Titanium chemistry (454 Life Sciences). Genomic sequences were compared to the NCBI non-redundant (Nr) database using the blastn algorithm in order to find SuMu bacteriophage genes in *H. parasuis* 34086b.

### SDS-PAGE analysis

For 2-D electrophoresis, 1000 μg (determined by bicinchoninic acid (BCA) assay) (Sigma) of bacteriophage preparation or protein precipitated from BHI media control were mixed with an equal volume of 100 mM MgCl_2_, plus one-tenth volume of DNase I (Sigma), and one-tenth volume of RNase A (Sigma). The mixture was incubated on ice for 1 h; then an equal volume of freshly prepared 40% TCA in acetone was added and the mixture was stored at −20°C overnight [43,44]. The mixture was centrifuged at 17,900 × *g* for 10 min at room temperature and subsequently washed twice with a solution containing 0.2% dithiothreitol (Sigma) in acetone [43]. The precipitate was air-dried and resuspended in 200 μl immobilized pH gradient (IPG) buffer. The solution was sonicated lightly to disperse the pellet. The 2D-Quant Assay (Amersham Biosciences) was performed to determine the concentration of the TCA-precipitated lysate.

A ten-well NuPAGE precast 4-12% gradient Bis-Tris gel (Invitrogen) was run after applying 10 μg/well of (TCA)-precipitated sample in order to check the concentration of the protein on the Coomassie-stained gel and for possible degradation of the sample. The prestained protein standard was BenchMark, 10–200 kDa (Invitrogen). Gels were run according to manufacturer’s instructions, stained with Coomassie Brilliant Blue and destained as described [45].

The following modifications of operation of a Multiphor II isoelectric focusing (IEF) system (Amersham Pharmacia Biotech, Upsula, Sweden) were implemented for the first dimension conditions of the 2-D electrophoresis procedure. A 200 μg sample of the TCA-precipitated bacteriophage pellet in IPG buffer containing 2.5 mM tributylphosphine [46] and 0.1% DTT was applied to a 3–10 pH Immobiline dry strip (Amersham Pharmacia Biotech). The following protocol was used: 1) the strip was rehydrated at 30°C for 12 h; 2) voltage was adjusted to increasing values as follows: 500 V at 20°C for 90 min; 1000 V at 20°C for 90 min; 2000 V at 20°C for 60 min; 4000 V at 20°C for 60 min; 6000 V at 20°C for 90 min; 8000 V at 20°C for 7 h for a total of 35,700 Vh. The standards for the first dimension were IEF mix 3.6-9.3 (Sigma). For the second dimension, the IEF strips were equilibrated in 1% dithiothreitol, then in 5% iodoacetamide for 25 min to alkylate the sulfhydryl groups before being applied to a NuPAGE 4-12% gel with an IPG well (Invitrogen). Second dimension electrophoresis conditions and staining were done as described for the 1-D gels.

### Mass spectrometry and data analysis

Mass spectrometry was performed at the Iowa State University Protein Facility and the Iowa State University Proteomics Facility. For matrix-assisted laser desorption/ionization-time of flight (MALDI-TOF), three plugs of the band of interest from a 1-D gel were picked with a blunt-cut 20 gauge needle and deposited in a 96-well tray of an automated digester according to the procedures recommended by ProGest (Genomic Solutions). Plugs were subjected to limited trypsin (Invitrogen) digestion. A C18 ZipTip (Millipore) was wetted with 10 μl of 70% acetonitrile in water. The solvent was discarded and the tip was washed three more times. Subsequently, the C18 ZipTip was washed twice with water and once with 1.0% TFA. The digested sample (10 μl) was aspirated and dispensed 30 times. The C18 ZipTip was rinsed three times with 0.1% TFA and then dried. Matrix solution (20 mg/ml α-cyano-4-hydroxycinnamic acid in 50% acetonitrile/water/0.1% TFA) (0.5 μl) was dispensed onto a MALDI plate. Samples were mixed with the matrix solution on the target plate. Dried spots were subjected to mass peptide fingerprinting using on a Voyager System 6075 (PE Biosystems, Foster City, CA). Spectra were analyzed with the MSFit tool of the Protein Prospector program [47] at http://www.prospector.ucsf.edu/prospector, using taxonomy filters for microorganisms or *Haemophilus*.

Tandem mass spectrometry (MS/MS) analysis was performed using a QSTAR XL quadrupole TOF mass spectrometer (ABI/MDS Sciex, Foster City, CA) equipped with an oMALDI ion source [48]. Gel plugs containing selected bands (1-D gel) or spots (2-D gel) were digested with sequencing-grade trypsin (Promega, Madison, WI) in ammonium bicarbonate buffer at 37°C overnight. Samples were loaded on target plates as above. All spectra were processed by MASCOT (MatrixScience, London, UK) database search. Peak lists were generated by Analyst QS (ABI/MDS Sciex, Foster City, CA) and were used for MS/MS ion searches. Typical search parameters were as follows: Maximum missed cleavage setting was 1.0; the fixed modification setting was carboxyamidomethyl cysteine with a variable modification of oxidation of methionine. Peptide mass tolerances were +/− 100 ppm. Fragment mass tolerances were +/− 1 Da or +/− 2 Da. Protein molecular weights searched +/− 10–20 kDa from observed molecular weights and no restrictions on pI were applied. Protein identification was based on the probability based MOWSE (MOlecular Weight SEarch) Score (*p* < 0.05) and % coverage of peptide [47]. Sequences were aligned with those in the database using blastp and bl2seq from NCBI; the degree of similarity was expressed as *E*[15,28]. An *E*-value or Expection value represents the number of different alignments with scores equivalent or better than the sum of substitution and gap scores that is expected to occur in a database search by chance. The lower the *E*-value, the more significant is the score and alignment between the two proteins.

### Accession numbers and files

The DNA sequence for the bacteriophage reported here was deposited in GenBank, Accession No. JF832915. Mass spectrometry data in the mzML format was supplied as additional file 4: P1.mzML, additional file 5: P2.mzML, additional file 6: P3.mzML, additional file 7: P4.mzML, additional file 8: P5.mzML, additional file 9: P6.mzML, additional file 10: P7.mzML, additional file 11: P8.mzML, additional file 12: P9.mzML, additional file 13: P10.mzML, additional file 14: P11.mzML, additional file 15: P12.mzML, additional file 16: P13.mzML, additional file 17: P14.mzML, additional file 18: P15.mzML, additional file 19: P16.mzML, additional file 20: P17.mzML; additional file 21: P18.mzML. The LCMS-Lysate.mzML file was very large so four jpg files (additional files 22-25, JPEG gp36, pages 1-4) were used to present the gp36 tail fiber mass spectrometry data. Tandem mass spectrometry data was also deposited in the ProteomeExchange (http://proteomexchange.org/) through the proteomics identifications database (PRIDE) website (http://www.ebi.ac.uk/pride/) (accession numbers 22479, 22480, 22482, 22501–22506) [49,50]. The data was converted using PRIDE Converter [51] (http://pride-converter.googlecode.com).

## Abbreviations

1-D and 2-D, 1- and 2- dimensional; ACT, Artemis Comparison Tool; Bp, Base pair; β-NAD, β-Nicotinamide adenine dinucleotide; CDS, Coding sequence; EDTA, Ethylenediaminetetraacetic acid; HCl, Hydrodrochloric acid; IPG, Immobilized pH gradient; IEF, Isoelectric focusing; MALDI-TOF, Matrix-assisted laser desorption/ionization-time of flight; MWCO, Molecular Weight Cut Off; NAHMS, National Animal Health Monitoring System; NCBI, National Center for Biotechnology Information; Nr, Non-redundant; ORF, Open reading frame; PCR, Polymerase chain reaction; PRIDE, Proteomics identifications database; SDS-PAGE, Sodium dodecyl sulfate polyacrylamide gel electrophoresis; SEW, Segregated Early Weaning; TCA, Tricholoroacetic acid; TFA, Trifluoroacetic acid.

## Competing interests

The authors declare they have no competing interests.

## Authors’ contributions

ESZ discovered the bacteriophage and did the molecular genomic and proteomic studies, LBT was involved in drafting the manuscript and revising it critically and served as PhD mentor for ESZ, DOB was involved in the bioinformatics aspects. All authors read and approved the final manuscript.

## Supplementary Material

Additional file 1 **Table S1.** Comparison of bacteriophage SuMu proteins identified in this study to homologous to enterobacteriophage Mu proteins; comparison of amino acid lengths and % homology of 17 bacteriophage SuMu proteins to enterobacteriophage Mu proteins, *E*-values are given. Click here for file

Additional file 2 **Table S2.** Comparison of SuMu proteins identified in this study to homologous Mu-like proteins; comparison of amino acid lengths and % homology of 25 bacteriophage SuMu proteins to Mu-like bacteriophage proteins, *E*-values are given. Click here for file

Additional file 3 **Table S3.** Homologs of bacteriophage SuMu from 1-D and 2-D SDS-PAGE gels identified by mass spectrometry; identification of 20 homologs of bacteriophage SuMu using mass spectrometry; scores, accession numbers, predicted MW/pI, number of peptides matched, % amino acid coverage are shown. Click here for file

Additional file 4 **P1.mzML** YP_239811, Phage tape measure protein, TP901 family, *Staphylococcus aureus* phage 2638A (Siphoviridae). Click here for file

Additional file 5 **P2.mzML** YP_004324195, Tail sheath monomer, *Synechococcus* phage S-SSM7 (Myoviridae). Click here for file

Additional file 6 **P3.mzML** YP_249019, Tail fiber protein, *H. influenzae* 86-028NP, similar to *Haemophilus* phage HP1 (Myoviridae). Click here for file

Additional file 7 **P6.mzML** P44242**,** Defective tail fiber protein, *H. influenzae* Rd KW20 Mu-like prophage FluMu (Myoviridae). Click here for file

Additional file 8 **P9.mzML** P44225**,** gp29, DUF935, portal protein, *H. influenzae* Rd KW20 Mu-like prophage FluMu (Myoviridae). Click here for file

Additional file 9 **P10.mzML** P44236**,** gp42, putative tape measure protein, *H. influenzae* Rd KW20 Mu-like prophage FluMu (Myoviridae). Click here for file

Additional file 10 **P12.mzML** EGT81757, Prophage integrase, *H. haemolyticus* M21639. Click here for file

Additional file 11 **P15.mzML** P96343, DNA transposition protein B (transposase), *H. influenzae* Rd KW20 Mu-like prophage FluMu (Myoviridae). Click here for file

Additional file 12 **P16.mzML** NP_873073, gp32, Mu I protein, *H. ducreyi* 35000HP, Mu-like prophage (Myoviridae). Click here for file

Additional file 13 **P17.mzML** P46496, DNA binding protein Ner, *H. influenzae* Rd KW20 Mu-like prophage FluMu (Myoviridae). Click here for file

Additional file 14 **P20.mzML** NP_463477, COG3941, putative tape measure protein, Mu-like prophage protein, *Listeria* phage A118 (Siphoviridae). Click here for file

Additional file 15 **P21.mzML** YP_001604090, Tail sheath protein, *Synechococcus* phage syn9, T4-like (Myoviridae). Click here for file

Additional file 16 **P22.mzML** YP_398561, Phage-related minor tail protein; pfam10145, *Clostridium* phage c-st (Myoviridae). Click here for file

Additional file 17 **P23.mzML** P44227**,** gp34 (gpT) major head subunit, *H. influenzae* Rd KW20 Mu-like prophage FluMu (Myoviridae). Click here for file

Additional file 18 **P24.mzML** Q01259**,** gp30, Mu F protein, Enterobacteriophage Mu (Myoviridae), Mu-like. Click here for file

Additional file 19 **P25.mzML** O05069, Transposase A, *H. influenzae* Rd KW20 Mu-like prophage FluMu (Myoviridae). Click here for file

Additional file 20 **ESI-Sample003.mzML** P10927**,** Baseplate wedge protein 9, *E. coli* enterobacteria phage T4 (Myoviridae). Click here for file

Additional file 21 **ESI-Sample004.mzML** P03744**,** gp37 long tail fiber, receptor recognizing protein, *E. coli* enterobacteria phage T4 (Myoviridae) and Q9T1X2 Lysozyme/muramidase/endolysin, Enterobacteriophage Mu (Myoviridae). Click here for file
